# Systemic administration of choline acetyltransferase decreases blood pressure in murine hypertension

**DOI:** 10.1186/s10020-021-00380-6

**Published:** 2021-10-21

**Authors:** Andrew Stiegler, Jian-Hua Li, Vivek Shah, Tea Tsaava, Aisling Tynan, Huan Yang, Yehuda Tamari, Michael Brines, Kevin J. Tracey, Sangeeta S. Chavan

**Affiliations:** 1 Institute of Bioelectronic Medicine, The Feinstein Institutes for Medical Research, Northwell Health, 350 Community Drive, Manhasset, NY 11030 USA; 2 Donald and Barbara Zucker School of Medicine at Hofstra/Northwell, 350 Community Drive, Manhasset, NY 11030 USA; 3 Circulatory Technology, Inc, 21 Singworth St, Oyster Bay, NY 11771 USA; 4 The Elmezzi Graduate School of Molecular Medicine, Northwell Health, 350 Community Drive, Manhasset, NY 11030 USA

**Keywords:** Hypertension, Acetylcholine, Choline acetyltransferase, PEG-ChAT

## Abstract

Acetylcholine (ACh) decreases blood pressure by stimulating endothelium nitric oxide-dependent vasodilation in resistance arterioles. Normal plasma contains choline acetyltransferase (ChAT) and its biosynthetic product ACh at appreciable concentrations to potentially act upon the endothelium to affect blood pressure. Recently we discovered a T-cell subset expressing ChAT (T_ChAT_), whereby genetic ablation of ChAT in these cells produces hypertension, indicating that production of ACh by T_ChAT_ regulates blood pressure. Accordingly, we reasoned that increasing systemic ChAT concentrations might induce vasodilation and reduce blood pressure. To evaluate this possibility, recombinant ChAT was administered intraperitoneally to mice having angiotensin II-induced hypertension. This intervention significantly and dose-dependently decreased mean arterial pressure. ChAT-mediated attenuation of blood pressure was reversed by administration of the nitric oxide synthesis blocker l-nitro arginine methyl ester, indicating ChAT administration decreases blood pressure by stimulating nitic oxide dependent vasodilation, consistent with an effect of ACh on the endothelium. To prolong the half life of circulating ChAT, the molecule was modified by covalently attaching repeating units of polyethylene glycol (PEG), resulting in enzymatically active PEG-ChAT. Administration of PEG-ChAT to hypertensive mice decreased mean arterial pressure with a longer response duration when compared to ChAT. Together these findings suggest further studies are warranted on the role of ChAT in hypertension.

**Supplementary Information:**

The online version contains supplementary material available at 10.1186/s10020-021-00380-6.

## Background

Approximately 100 million adults in the United States and more than 1 billion individuals worldwide have hypertension (Mozaffarian et al. 2015), which is a major risk factor for cardiovascular disease, kidney disease, stroke and cognitive impairment (Pugh et al. 2019; González et al. 2018; Zhou et al. 2018; World Health Organization 2013). Abnormalities in the renin-angiotensin system (RAS) have been implicated in a number of forms of clinically significant elevated blood pressure, including essential hypertension (Crowley et al. 2006). Currently available therapies to treat hypertension are inadequate, because 75% of medicated hypertensive patients continue to have increased blood pressure (Whelton et al. 2017). Therefore, new therapeutic strategies for hypertension are urgently needed.

Changes in blood vessel diameter produce changes in blood pressure via active vasoconstriction or vasodilation, which is a major physiological mechanism to regulate blood pressure. Endothelial cells lining resistance arterioles are stimulated to mediate vasodilation when exposed to acetylcholine (ACh) which induces the release of nitric oxide (NO), termed endothelium derived relaxing factor or EDRF (Higashi et al. 2012; Mónica et al. 2016). ACh interacts with muscarinic receptors expressed on endothelial cells, and receptor activation induces phosphorylation of endothelial nitric oxide synthase, the rate limiting enzyme in the biosynthesis of NO (Dimmeler et al. 1999; Palmer et al. 1988; Zhou et al. 2005). NO produced by endothelial cells diffuses to adjacent smooth muscle cells, interacts with the heme group in souble guanylate cyclase to stimulate biosynthesis of cGMP, a secondary messenger. CGMP in turn activates signaling cascades to release calcium ion from intracellular stores which produces relaxation of smooth muscle cells and decrease in blood pressure.

While a number of studies have shown that lymphocytes within the circulation (Kawashima and Fujii 2004) as well as the endothelium itself (Kawashima et al. 1990) can produce ACh and therefore potentially regulate EDRF, serum also contains significant concentrations of ACh (Fujii et al. 1995). Additionally, serum also contains soluble ChAT, the enzyme responsible for ACh biosynthesis, as well as the cholinestarases which degrade ACh (Vijayaraghavan et al. 2013). Recent study has shown that within the serum (and cerebral spinal fluid) ACh concentration is maintained at a steady state balance via a specific ratio of ChAT to cholinesterases, which varies in different disease states (Vijayaraghavan et al. 2013).

We previously identified a role for an ACh-producing T cell subset, termed T_ChAT_, in regulating blood pressure (Olofsson et al. 2016), as genetic ablation of ChAT in those T cells produced hypertension (Olofsson et al. 2016). Prior studies have shown that the activation of T cells results in the release of ChAT into the circulation (Vijayaraghavan et al. 2013; Fujii et al. 2017a; Awwad et al. 2014; Speziale et al. 2017). Additionally, it has been observed that decreased ChAT levels and ACh production by lymphocytes occurs in spontaneously hypertensive rats (Fujimoto et al. 2001). Because ACh stimulates EDRF, and increasing serum ACh levels decreases blood pressure in hypertension (Liu et al. 2017; Lataro et al. 2015), we reasoned direct administration of ChAT could tip the balance for increased circulating ACh and therefore decrease blood pressure in hypertension. We tested this hypothesis in mice by using a continuous infusion of angiotensin II to produce hypertension via its direct effect on the handling of sodium by the kidney (Crowley et al. 2006), while simultaneously administering ChAT and thereby increasing ACh synthesis.

## Materials and Methods

### Animals

All procedures with experimental animals were approved by the Institutional Animal Care and Use Committee and the Institutional Biosafety Committee of the Feinstein Institutes for Medical Research, Northwell Health, Manhasset, NY in accordance with NIH guidelines. Animals were maintained at 25 °C on a 12 h light–dark cycle with free access to food and water. 12-week old male C57BL/6 mice implanted with HD-X10 blood pressure telemeters were obtained from The Jackson Laboratory (Bar Harbor, ME). Mice were singly housed after implanting blood pressure telemeters. ChAT and PEG-ChAT were administered intraperitoneally.

### Blood pressure, temperature and activity measurements

Mice were induced under 2.5% isoflurane and implanted with HD-X10 blood pressure telemeters (Data Sciences Inc., New Brighton, MN) according to the manufacturer’s instructions. Briefly, mice were anesthetized and placed in dorsal recumbency. A small incision was made in the neck and the left carotid artery was isolated from the surrounding tissue. A hollow catheter was placed in the left carotid artery and advanced to the aortic arch. Once in place, the catheter was secured to the carotid artery with a suture knot. The telemeter body was placed in a subcutaneous pocket on the animal’s flank, and the animal was recovered for 7 days before blood pressure measurements were taken. Surgeries were performed by JAX Surgical Services (The Jackson Laboratory, Bar Harbor, ME).

### Angiotensin II administration

Osmotic pumps (Alzet model 1004, Cupertino CA, 95014) were loaded with angiotensin II (Fisher Scientific) solution to produce the desired flow rate (700 ng/kg/min) according to the manufacturer’s instructions and incubated in sterile saline at 37 °C for 48 h before implantation. To implant the pump, mice were induced under 2.5% isoflurane anesthesia and a 1 cm midscapular incision was made. The pump was inserted into the midscapular incision and advanced away from the incision site. The incision was closed with a wound clip or sutures. After 24 h of recovery, blood pressure measurements resumed. After 28 days post-implant, the implants were removed according to the manufacturer’s instructions.

### Production of recombinant ChAT protein and ChAT PEGylation

Recombinant human ChAT corresponding to residue 119–748 (EC2.3.1.6, UniProt 28329-3) with a *N*-histidine tag was expressed in *E. coli* BL21-Gold (DE3) cells. Bacteria were cultured in 2xYT media to an A600 of 0.9. IPTG was added to a final concentration of 3 mM to induce recombinant ChAT production. Five hours later, cells were harvested, re-suspended in cold binding buffer (containing 20 mM Tris, 1% X-100, pH 8.5) and subjected to sonication at 4 °C. Supernatant was collected by centrifugation at 13,000×*g* for 20 min and applied onto a 5-ml high affinity Ni-charged column pre-equilibrated with binding buffer. Following sequential washings with the binding buffer, washing buffer 1 (20 mM Tris, 10 mM imidazole, 0.5 M NaCl, pH 8.0), and buffer 2 (0.2× phosphate buffered saline (PBS), 10% glycerol, pH 7.5), the recombinant histidine-tagged ChAT protein was eluted with 0.5 M imidazole, 10% glycerol, in 0.2× PBS. The recombinant ChAT was further purified by dialysis at 4 °C in buffer containing 0.2× PBS, 10% glycerol and 0.5 mM TCEP (tris(2-carboxyethyl)phosphine), and extensive Triton X-114 extraction to remove contaminating endotoxins. PEGylation was performed using an unbranched amine-reactive MS (PEG)12 reagent (Thermo Scientific), in a buffer containing 10% glycerol, 0.2× PBS, 0.5 mM TCEP at a molar ratio of ChAT protein to MS (PEG)12 of 1–200. Following PEGylation, the protein was then dialyzed in buffer and further extracted with Triton X-114 to remove contaminating endotoxins.

### Enzyme activity assay

Activity of recombinant and PEGylated recombinant ChAT was analyzed using an adapted version of the colorimetric assay developed by Kumar et al. (2016). Briefly, ChAT and its substrates choline and acetyl-coenzyme A were incubated at 37 °C for 15 min in a total volume of 100 μL containing buffer containing 10 mM Tris–HCl, 150 mM NaCl, 1 mM EDTA, and 0.05% Triton X-100 (final concentrations 1.5 µg/mL ChAT, 150 µM choline, 150 µM acetyl-coenzyme A). After incubation, a cocktail containing choline oxidase, 4-aminoantipyrine, phenol, and horseradish peroxidase (HRP) was added (cocktail concentration 10 U/mL choline oxidase, 3 mM 4-aminoantipyrine, 6 mM phenol, 50 U/mL HRP). Red color developed in proportion with the remaining choline in the reaction mixture is quantitated spectrophotometrically at 500 nm. By subtracting the remaining choline in wells containing ChAT from the choline remaining in a control well without any enzyme, the reaction rate is calculated.

### Data analysis

**Fig. 1:**
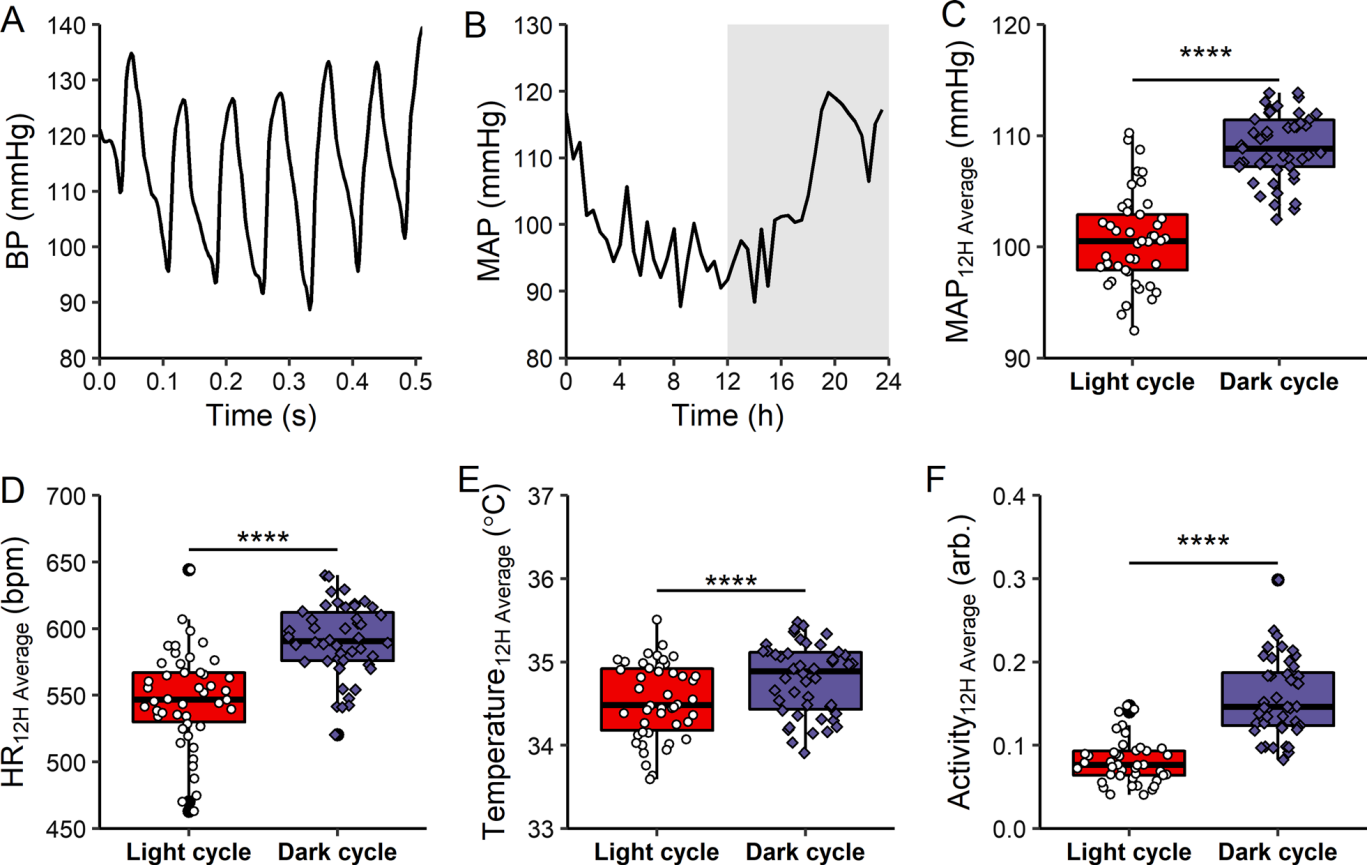
Telemetry system for analyzing short-term and long-term changes in blood pressure. **A** representative blood pressure wave tracing from a single telemeter-implanted mouse. BP is measured at 500 Hz. **B** representative 24 h tracing of MAP using the same telemeter system. From the blood pressure wave, MAP is calculated every 10 s. These 10 s bins are averaged over 30 min. Gray background indicates dark cycle when mice are active. **C** average normotensive MAP, **D** average normotensive HR, **E** average normotensive body temperature and **F** average normotensive activity. MAP, HR, temperature and activity are analyzed during 12 h of dark cycle and light cycle. Data are represented as individual mouse data points with mean ± SEM. n = 44, ****p < 0.0001, paired t-test

**Fig. 2:**
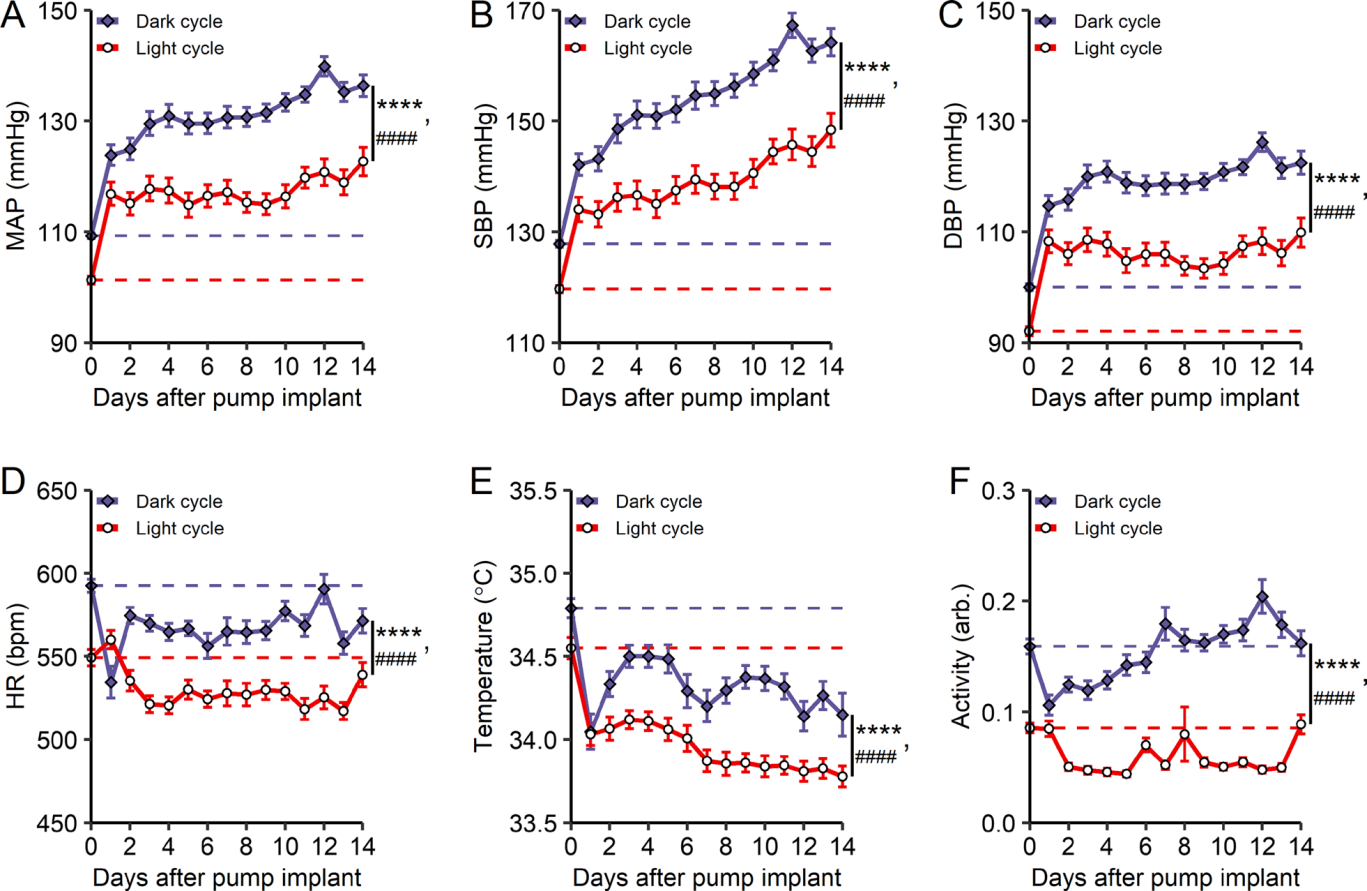
Angiotensin II-induced hypertension model. **A**–**F** change in MAP, SBP, DBP, heart rate, body temperature, and activity levels during angiotensin II infusion. Dark cycle and light cycle average values were collected prior to and during angiotensin II infusion. Osmotic pumps were implanted on day 0. Data is collected continuously and averaged over the 12 h light and dark cycles separately on each day. Day 0 values represent an average of 3 days prior to implantation of osmotic pumps delivering angiotensin II. Dashed lines represent data in normotensive animals in light and dark cycles. **A** MAP, **B** SBP and **C** DBP significantly increase during angiotensin II infusion. Data are represented as mean ± SEM, n = 41–50, ****p < 0.0001, light cycle vs. dark cycle, ^####^p < 0.0001, over time, two-way ANOVA with Sidak’s multiple comparison test. **D** HR decreases during angiotensin II infusion. Dark cyle HR remains significantly elevated compared to light cycle HR. Data are represented as mean ± SEM, n = 41–50, ****p < 0.0001, light cycle vs. dark cycle, ^####^p < 0.0001, over time, two-way ANOVA with Sidak’s multiple comparison test. **E** body temperature decreases during angiotensin II infusion. Dark cycle body temperature remains significantly elevated compared to light cycle body temperature. Data are represented as mean ± SEM, n = 41–50, ****p < 0.0001, light cycle vs. dark cycle, ^####^p < 0.0001, over time, two-way ANOVA with Sidak’s multiple comparison test. **F** activity levels decrease during angiotensin II infusion, with dark cycle activity levels remaining significantly elevated compared to light cycle activity levels. Data are represented as mean ± SEM, n = 41–50, ****p < 0.0001, light cycle vs. dark cycle, ^####^p < 0.0001, over time, two-way ANOVA with Sidak’s multiple comparison test

**Fig. 3:**
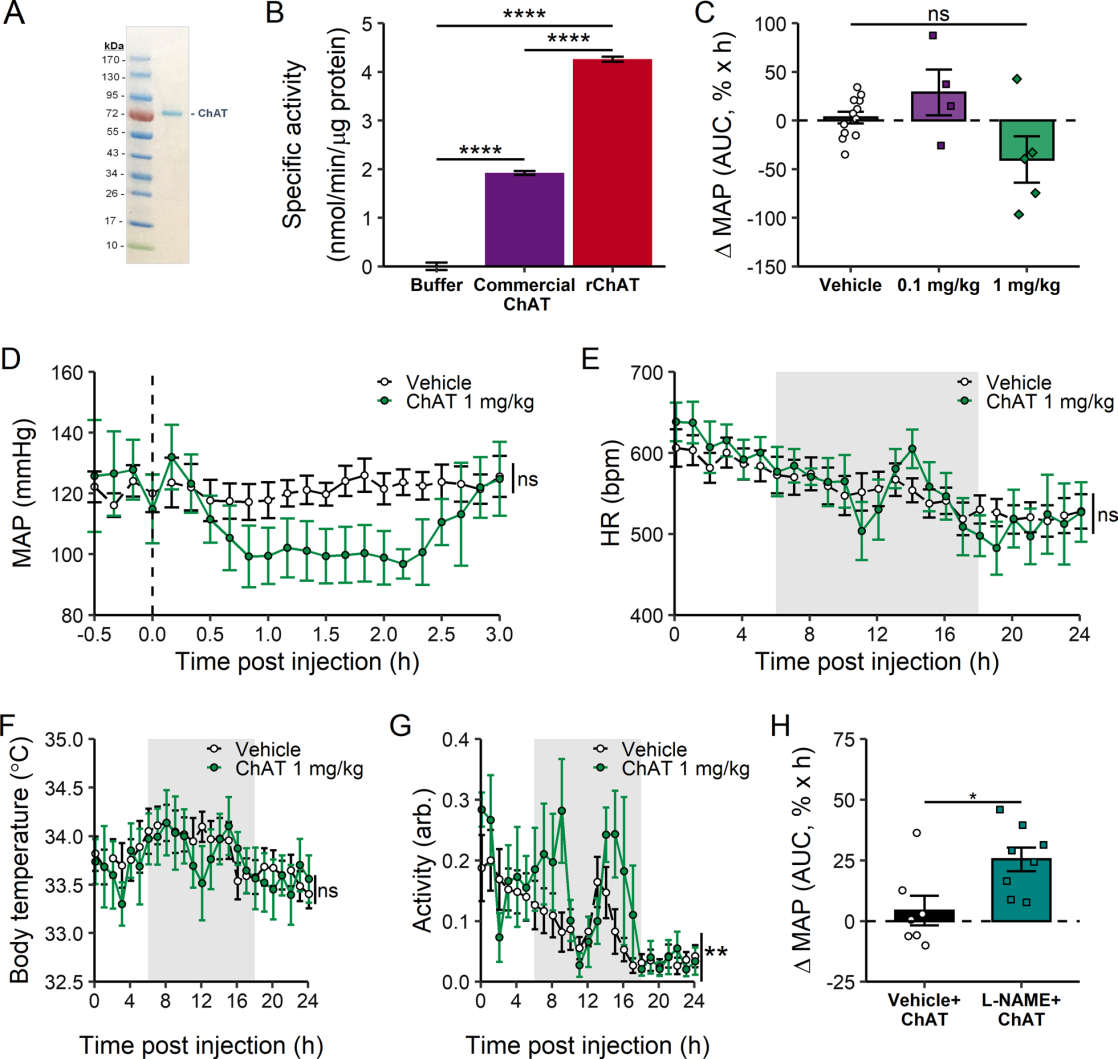
ChAT administration decreases blood pressure. **A** SDS-PAGE of purified recombinant human ChAT. As predicted from sequence, ChAT has a molecular weight of ~ 72 kD. **B** specific activity of recombinant ChAT (rChAT) and commercially available ChAT. Data represented as one assay performed in triplicate. ****p < 0.0001, one-way ANOVA with Dunnett’s correction, **C** normalized MAP in the 3 h period after ChAT administration. MAP is normalized to the 30-min period before ChAT or vehicle administration, and area under the curve is calculated from a baseline of 100%. n = 5–11. ns, not significant, one-way ANOVA with Dunnett’s multiple comparison test. **D** MAP after ChAT administration at time 0. Data are averaged every 20 min and represented as group mean ± SEM, n = 5–11. ns: not significant, two-way ANOVA with Sidak’s multiple comparisons test. **E** HR, **F** body temperature and **G** activity scores in the 24 h period post-ChAT administration. Gray background indicates dark cycle. Data are averaged every hour and represented with lines as group mean ± SEM. *ns* not significant, **p < 0.01, two-way ANOVA with Sidak’s multiple comparison test. **H** change in normalized MAP. MAP is normalized to the 30-min period before L-NAME or vehicle pre-treatment. After normalization, area under the curve for 1 h post-ChAT administration is calculated from a baseline of 100%. Negative values correspond to decrease in MAP. n = 7–8. *p < 0.05, t-test

**Fig. 4:**
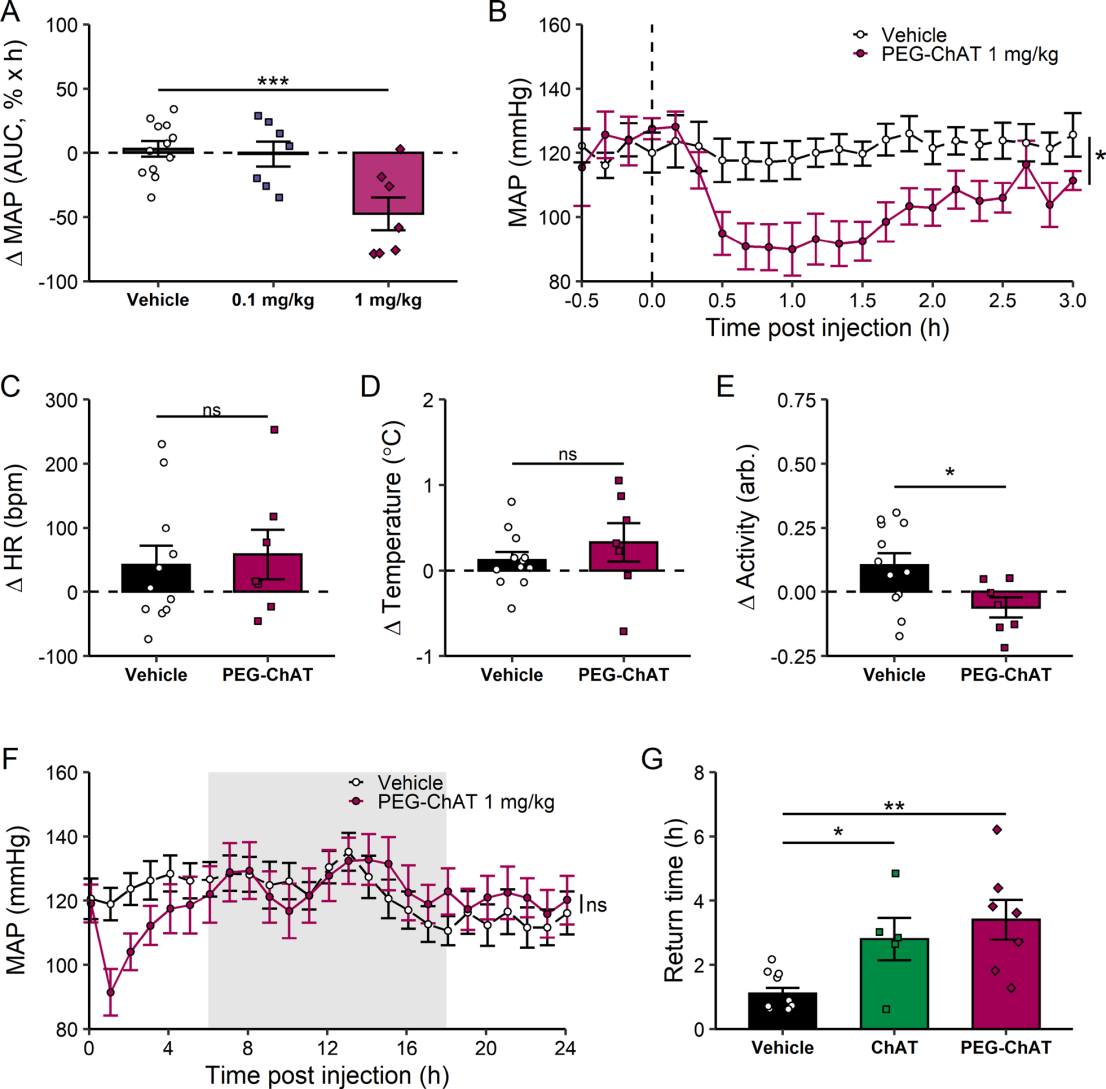
PEGylation increases duration of ChAT-induced blood pressure decrease. **A** change in normalized MAP. MAP is normalized to the 30-min period before PEG-ChAT or vehicle administration, and area under the curve is calculated from a baseline of 100% in the 3 h post-PEG-ChAT administration. Negative values correspond to decrease in MAP. n = 7–11. ***p < 0.001, 1 mg/kg vs. vehicle. One-way ANOVA with Dunnett’s multiple comparison test. **B** MAP in 3 h post-PEG-ChAT administration. MAP was compared between PEG-ChAT and vehicle groups at each time point post-administration. n = 7–11. *p < 0.05, two-way ANOVA with Sidak’s multiple comparison test. **C**–**E** changes in **C** heart rate, **D** temperature and **E** activity after PEG-ChAT administration. Change is calculated as the baseline (30 min pre-administration) subtracted from the average data in the 3 h post-administration period. Data are represented as individual mouse data points, mean ± SEM. n = 7–11. *ns* not significant, *p < 0.05, t-test. **F** MAP in the 24 h post-PEG-ChAT administration. Gray background indicates dark cycle. Data are averaged every hour and represented with lines as group mean ± SEM. n = 7–11. ns: not significant, two-way ANOVA with Sidak’s multiple comparison test. **G** time course responses to ChAT and PEG-ChAT. Baseline was defined as 30-min average MAP prior to administration. Individual post-administration MAP was normalized to individual pre-administration MAP, and return time was calculated for each animal. Return time was defined as the minimum time after administration where mice returned to baseline MAP for a 10-min average period. Data are represented as individual mouse points, with bars with mean ± SEM. n = 5–11. *p < 0.05, **p < 0.01, one-way ANOVA with Dunnett’s multiple comparison test

Data were analyzed using Graphpad Prism 7.0, Microsoft Excel, DSI Ponemah 6.4, and R. Blood pressure was sampled at 500 Hz and aggregated into 10 s bins to calculate SBP, DBP, MAP, and heart rate. Temperature and activity data were separately averaged over 10 s bins. When averaged over 12 h, data are first binned in 10 s bins, and 12 h of bins are averaged together (Figs. 1, 2). When averaged every 20 min, data are first binned and 20 min of bins are averaged together (Figs. 3D, 4B). When averaged hourly, data are first binned in 10 s bins, and 60 min of bins are averaged together (Figs. 3E–G, 4F, Additional file 1: Fig. S7). In all plots, data are presented as mean ± SEM. Plots were generated using the R packages *ggplot2* and *patchwork*. Groups were compared using one-way ANOVA with Dunnett’s correction, two-way ANOVA with repeated measures with Sidak’s correction, or paired t-test where indicated. P values < 0.05 were considered significant.

## Results

### Short- and long-term changes in physiological parameters in hypertensive mice

A widely used murine preclinical model of essential hypertension is administration of angiotensin II (Ang II) (Simon et al. 1995; Edgley et al. 2001). Here we measured blood pressure in conscious and unrestrained mice using radiotelemetry monitoring of an intraarterial transducer in the aortic arch, which generated high-fidelity blood pressure recordings (Fig. 1A, B). Because mice are nocturnal, mean arterial blood pressure (MAP) during the dark cycle (active cycle for mice) is significantly elevated as compared to the light cycle (MAP: dark cycle 111 ± 0.6 mmHg vs. light cycle 102 ± 1.1 mmHg, p < 0.0001, Fig. 1C, Table 1). Heart rate (HR), core body temperature and activity were also monitored during dark and light cycles using radiotelemetry, revealing significant increases in HR (HR: dark cycle 586 ± 5 bpm vs. light cycle 541 ± 6 bpm, p < 0.0001, Fig. 1D), temperature (body temperature: dark cycle 34.7 ± 0.06 °C vs. light cycle 34.0 ± 0.05, p < 0.0001, Fig. 1E), and activity (activity: dark cycle 0.18 ± 0.01 abritary units vs. light cycle 0.07 ± 0.01 abritary units, p < 0.0001, Fig. 1F).

**Table 1:** Blood pressure in Angiotensin II-induced hypertension model

		Mean	95% CI	Range
MAP, mmHg				
Normotensive	Light cycle	101	(99.6–102.5)	92–120
	Dark cycle	109	(108.3–110.4)	102–123
Hypertensive	Light cycle	119	(115.8–122.5)	98–150
	Dark cycle	136	(133.6–139.0)	111–153
SBP, mmHg				
Normotensive	Light cycle	120	(118.3–120.9)	112–134
	Dark cycle	128	(126.7–129.3)	120–140
Hypertensive	Light cycle	144	(139.7–147.8)	119–172
	Dark cycle	163	(159.2–166.4)	131–185
DBP, mmHg				
Normotensive	Light cycle	92	(90.0–93.5)	82–112
	Dark cycle	100	(98.8–101.2)	92–114
Hypertensive	Light cycle	107	(103.5–110.1)	87–139
	Dark cycle	123	(120.4–125.7)	102–141

To induce hypertension, mice were implanted with osmotic pumps delivering Ang II (700 ng/kg/min). MAP increases significantly within 24 h of Ang II pump implantation during both light cycle and dark cycle (Fig. 2A). As observed in normotensive mice (Fig. 1C), MAP during Ang II infusion is significantly higher in the dark cycle (active) than the light cycle (Fig. 2A, Table 1). MAP continues to increase for at least eight days, rising to 136 ± 1.2 mmHg in the dark cycle (25% increase compared to normotensive), and 119 ± 1.5 mmHg in the light cycle (18% increase compared to normotensive) (Fig. 2A). Significant increases occur in both the systolic blood pressure (SBP) and diastolic blood pressure (DBP) (Fig. 2B, C and Table 1). Compared to normotensive baseline values, both SBP and DBP are increased approximately 25% in the dark cycle and 18% in the light cycle (Fig. 2B, C and Table 1). After Ang II infusion, heart rate decreases from 591 ± 4 bpm to 575 ± 5 bpm during the dark cycle and from 547 ± 5 bpm to 526 ± 4 bpm during the light cycle (Fig. 2D). Body temperature decreases from 34.8 ± 0.1 °C to 34.3 ± 0.1 °C during the dark cycle, and from 34.5 ± 0.1 °C to 33.9 ± 0.1 °C during the light cycle (Fig. 2E). Activity is increased in the dark cycle (from 0.16 ± 0.01 units vs 0.19 ± 0.01 units), but is decreased during the light cycle (from 0.04 ± 0.003 units in normotensive vs 0.06 ± 0.003 units in hypertensive, Fig. 2F).

### ChAT protein decreases blood pressure in angiotensin II-induced hypertension

To study the effects of ChAT administration in the context of hypertension, we expressed recombinant human ChAT protein in an *Escherichia coli* expression system. As expected from the cDNA sequence for the R isoform (UniProt P28329-3), purified ChAT has an observed migration on electrophoresis of approximately 72 kDa (Fig. 3A). To confirm recombinant ChAT protein is functionally active we measured enzyme activity by a colorimetric assay (Kumar et al. 2017) based on depletion of choline from the reaction. Recombinant purified ChAT protein exibits twofold higher specific activity as compared to a commercially available (MyBioSource) ChAT preparation (4.26 ± 0.04 nmol/min/µg protein *vs* 1.92 ± 0.04 nmol/min/µg protein) (Fig. 3B).

Recent clinical data indicates administration of anti-hypertensive agents at night optimizes blood pressure control and improves cardiovascular risk reduction (Hermida et al. 2019). Accordingly, we administered recombinant ChAT in the middle of the murine sleep cycle, which also corresponded to the time of day with the lowest blood pressure. In the Ang II-induced hypertension model, ChAT administration dose-dependently reduces Ang II-induced elevation of MAP (Fig. 3C), SBP (Additional file 1: Fig. S1A) and DBP (Additional file 1: Fig. S1B), when measured as area-under-the-curve compared to baseline blood pressures. A single intraperitoneal administration of ChAT (1 mg/kg) reduces average MAP as compared to vehicle control that lasts for 3 h post-administration (121 ± 4 mmHg vehicle, 107 ± 8 mmHg ChAT) (Fig. 3D).

As blood pressure is positively correlated with both HR and activity levels (Christofaro et al. 2017; Colangelo et al. 2020), we assessed changes in HR and activity scores post-ChAT administration. An immediate increase in HR is observed after either vehicle or ChAT administration as a result of intraperitoneal injection stress (Additional file 1: Fig. S2), eventually stabilizing at a level comparable to the vehicle control in the 24 h post-administration period (Fig. 3E). Unlike HR, body temperature responds slowly to stress and there is no difference in body temperature after ChAT or vehicle administration (Fig. 3F, Additional file 1: Fig. S3). As ChAT was administered in the middle of the light cycle (sleep cycle for mice), the animals are at their minimum activity levels prior to administration. Similar to changes in the HR, an initial increase in the activity levels is observed after injection stress. In the 24-h post-injection period activity levels were significantly increased with ChAT administration compared to vehicle administration (Fig. 3G, Additional file 1: Fig. S4).

The relatively unchanged HR and activity levels after ChAT administration are consistent with a mechanism of ACh-induced vasorelaxation (Rabelo et al. 2008). ACh acts on muscarinic receptors on endothelial cells, and produces arterial relaxation by activating endothelial nitric oxide synthase that produces NO (Dimmeler et al. 1999). Based on the hypothesis that ChAT administration increases endogenous ACh production to induce vasorelaxation, we reasoned that ChAT-induced blood pressure decrease is NO-dependent. To investigate this mechanism, we administered nitric oxide synthase inhibitor N**ω**-nitro-l-arginine methyl ester (L-NAME), and measured blood pressure following administration of ChAT. Pre-treatment with L-NAME significantly attenuates ChAT-mediated decrease in blood pressure (Vehicle vs L-NAME: 4.3 ± 6.1%MAP × h vs 25.4 ± 4.9%MAP × h, p = 0.019, Fig. 3H). Together, these results indicate administration of recombinant ChAT decreases blood pressure through a NO-mediated endothelium-dependent vasorelaxation mechanism.

### PEGylated ChAT retains activity and decreases blood pressure

To prolong the half life, ChAT was modified by covalently attaching repeating units of polyethylene glycol (PEG). This method is a useful strategy to enhance the pharmacokinetic properties of therapeutic proteins as it increases protein’s hydrodynamic radius and decreases immunogenicity and proteolysis (Jevševar et al. 2010; Veronese 2001; Simone 2008). Recombinant ChAT was pegylated using an amine-reactive PEG reagent to generate PEGylated ChAT (PEG-ChAT) with a molecular weight of approximately 95 kDa, a ~ 20 kD increase compared to recombinant ChAT (Additional file 1: Fig. S5A). The enzymatic activity of PEG-ChAT was confirmed using the colorimetric assay, indicating pegylated protein retains the activity to catalyze ACh production (Specific activity: 4.45 ± 0.03 nmol/min/µg ChAT vs 4.25 ± 0.04 nmol/min/µg PEG-ChAT) (Additional file 1: Fig. S5B). When given to mice, a single dose of PEG-ChAT in the middle of the light cycle (sleep cycle) reduces Ang II-induced elevation of MAP (vehicle vs 1 mg/kg PEG-ChAT: 3.16 ± 6.03%MAP AUC × h vs − 47.5 ± 12.6%MAP × h, p < 0.001, Fig. 4A), SBP and DBP (Additional file 1: Fig. S6A, B). A single intraperitoneal administration of PEG-ChAT (1 mg/kg) significantly reduces MAP as compared to vehicle administration that lasts for 3 h post-administration (MAP: 121 ± 4.5 mmHg vehicle vs 102 ± 4 mmHg PEG-ChAT 1 mg/kg, p < 0.05, Fig. 4B). PEG-ChAT fails to significantly increase HR (Fig. 4C) (30 ± 30 bpm for vehicle, 59 ± 39 bpm PEG-ChAT, Fig. 4C), and alter body temperature (0.12 ± 0.09 °C for vehicle, 0.33 ± 0.22 °C PEG-ChAT, Fig. 4D) in the 3-h period post-administration. Interestingly, PEG-ChAT decreases activity as compared to pre-administration levels (0.1 ± 0.05 units vehicle vs − 0.06 ± 0.04 units PEG-ChAT, p < 0.05, Fig. 4E), but this change in activity levels is transient and not seen in the 24 h period post-administration (Additional file 1: Fig. S7C). A single dose of PEG-ChAT decreases in MAP persist for 3 h (Fig. 4F), and also does not induce lasting changes in HR, body temperature or activity scores (Additional file 1: Fig S7A–C). Finally, to assess the duration of responses to ChAT and PEG-ChAT, we normalized individual post-administration MAP to individual pre-administration MAP, and calculated return time to pre-administration MAP for each animal. A significant increase in return time is observed after either ChAT or PEG-ChAT administration as compared to vehicle controls (Fig. 4G). An increased return time is observed after PEG-ChAT administration, indicating that PEG-ChAT maintains the lower MAP for a longer time (2.8 ± 0.7 h ChAT, 3.4 ± 0.6 h PEG-ChAT, Fig. 4G).

### Effect of ChAT depends on the time of administration

**Fig. 5:**
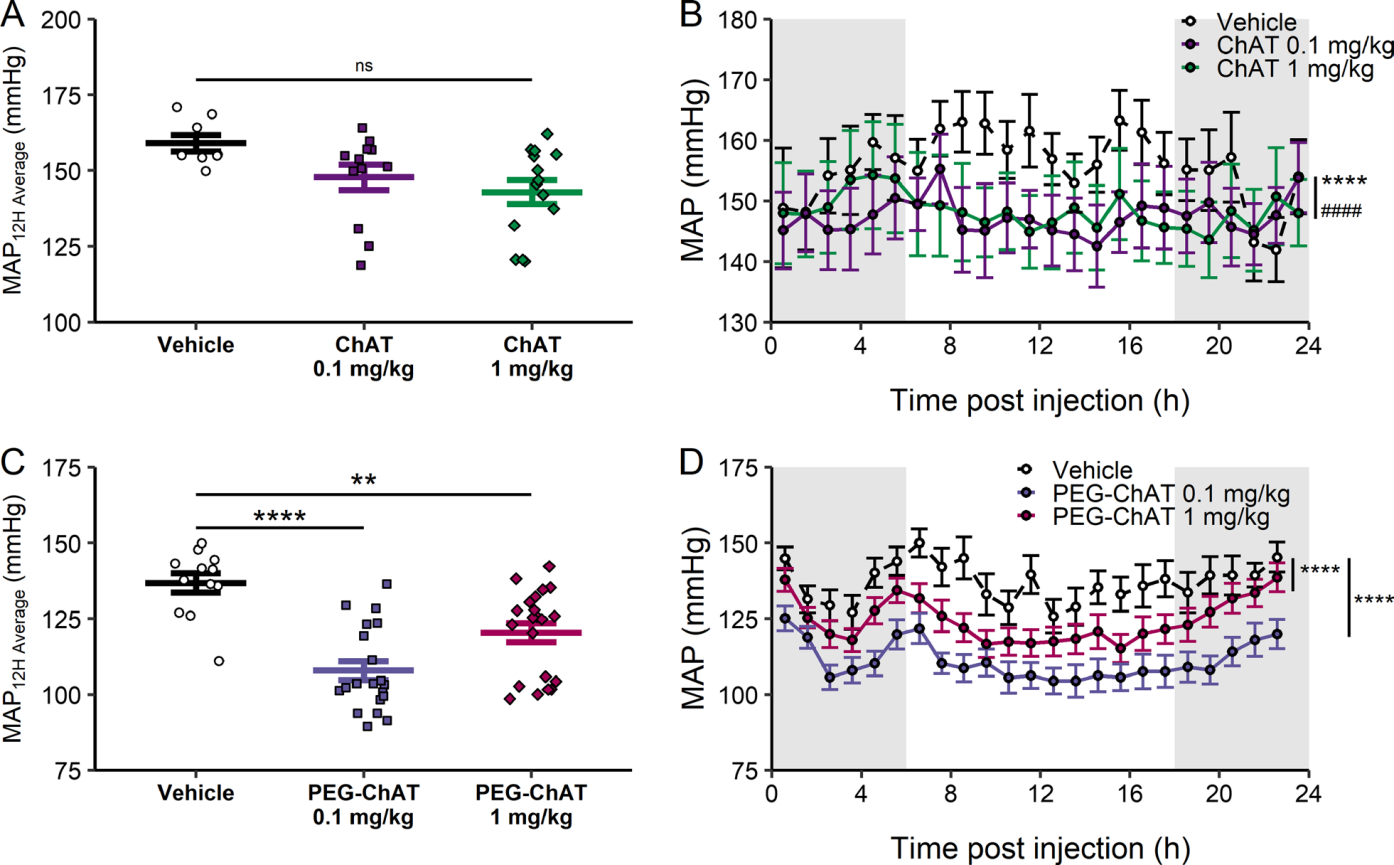
Administration of PEG-ChAT during dark cycle (active cycle) decreases blood pressure. **A** mean MAP in light cycle following a dark cycle administration of ChAT. ChAT was administered in the middle of the dark cycle (6 h), and MAP is averaged 6–18 h post-ChAT administration. Data are represented as individual mouse points with mean ± SEM. n = 8–15. *ns* not significant, one-way ANOVA with Dunnett’s multiple comparison test. **B** MAP in 24 h post-ChAT administration in dark cycle. Gray background indicates dark cycle. Data are averaged every hour and represented with lines as group mean ± SEM. n = 8–15. ****p < 0.0001, vehicle vs. 0.1 mg/kg ChAT, ^####^p < 0.0001, vehicle vs. 1 mg/kg ChAT, two-way ANOVA with Tukey’s multiple comparison test. **C** mean MAP in light cycle following a dark cycle administration of PEG-ChAT. PEG-ChAT was administered in the middle of the dark cycle (6 h), and MAP is averaged 6–18 h post-ChAT administration. Data are represented as individual mouse points with mean ± SEM. n = 12–21. **p < 0.01, ****p < 0.0001, one-way ANOVA with Dunnett’s multiple comparison test. **D** MAP in 24 h post-PEG-ChAT administration in dark cycle. Gray background indicates dark cycle. While there is no initial transient BP decrease, MAP is decreased during the next light cycle phase. Data are averaged every hour and represented with lines as group mean ± SEM. n = 12–21. ****p < 0.0001, vehicle vs. 0.1 mg/kg or 1 mg/kg PEG-ChAT, two-way ANOVA with Tukey’s multiple comparison test

Next, we assessed whether administration of ChAT or PEG-ChAT during the dark cycle when the blood pressure is at its peak levels, reduces MAP in Ang II-induced hypertensive mice. We administered recombinant ChAT or PEG-ChAT in the middle of the dark cycle (active cycle for mice), which corresponded to the time of the day with highest blood pressures (Fig. 1B). In the Ang II-induced hypertension model, a single administration of ChAT does not induce significant decreases in MAP when given during the dark cycle (Fig. 5A). However, when assessed over the entire 24-h period following ChAT administration, ChAT induces a persistant decrease in MAP (Fig. 5B). In contrast, administration of PEG-ChAT during the dark cycle induces a significant dose-dependent decrease in MAP that persists for at least 24 h (Fig. 5C, D). Interestingly, lower doses of PEG-ChAT induce larger changes in MAP (Fig. 5C, D). In the 12 h light cycle following active-cycle administration, MAP was decreased to near normotensive levels with a single administration of PEG-ChAT (Figs. 1C, 5D). Accordingly, a persistent decrease in SBP and DBP is observed after administration of PEG-ChAT in the dark cycle (Additional file 1: Fig. S8A, 8B).

## Discussion

Here we report that administration of PEG-ChAT to mice with Ang II-induced hypertension significantly attenuates increases in MAP, SBP and DBP without altering other physiological parameters including HR, temperature and activity. These findings reveal the previously unrecognized potential use of exogenous ChAT as an experimental therapeutic in hypertension. Chronic infusion of Ang II in experimental animals induces a gradual increase in blood pressure (Simon et al. 1995; Romero and Reckelhoff 1999) primarily via its effect on angiotensin receptor 1 in the kidney (Crowley et al. 2006) and is widely used as a preclinical model for studies of cardiovascular physiology (Lerman et al. 1979; Lohmeier 2012). As expected, we observed that infusion of Ang II over a 2-week period in mice causes the gradual development of hypertension with associated changes in HR, body temperature and activity scores.

Despite extensive studies on ACh-mediated vasorelaxation, its role as a therapeutic strategy for cardiovascular diseases, especially in hypertension is not well-characterized. Conventional hypertension therapies focus on strategies for attenuating the activity of the renin-angiotensin system or by decreasing sympathetic nerve activity, because adrenergic neurons mediate vasoconstriction and increased peripheral resistance. However, increased parasympathetic vagus nerve cholinergic activity has been correlated with an improvement in endothelial dysfunction and aortic stiffening in spontaneously hypertensive rats (Annoni et al. 2019). Choline administration also slows the progression of hypertension in preclinical models, possibly by enhancing vagus nerve activity (Liu et al. 2017). Because the serum half-life of ACh is short (on the order of minutes), and because the vasculature is innervated by adrenergic and not cholinergic nerve fibers, the discovery of ACh-producing T cells provided an important new insight into cholinergic mechanisms of NO-dependent vasorelaxation (Sheng and Zhu 2018). ChAT-expressing CD4 T cells (CD4 T_ChAT_) are required to maintain normotension in mice via a NO-dependent mechanism. Ablation of ChAT in CD4 T cells leads to chronic hypertension indicating that ChAT expression by T cells is required to deliver ACh to endothelium devoid of direct cholinergic innervation (Olofsson et al. 2016).

ChAT is present as both an intracellular cytosolic enzyme in cholinergic cells and within extracellular compartments, incuding human plasma (Vijayaraghavan et al. 2013). Previous study has shown that in humans, soluble ChAT within the plasma and cerebrospinal fluid maintains a steady-state equilibrium of extrasynaptic ACh in the presence of ACh degrading enzymes, acetylcholinesterase and butyrylcholinesterase (Vijayaraghavan et al. 2013). Our finding that ChAT attenuates elevated blood pressure provides a possible explanation for ACh-mediated vasorelaxation through continuous renewal of ACh in the vasculature by soluble ChAT, and thereby maintenance of cardiovascular homeostasis in the absence of direct innervation with the cholinergic nerve fibers. These findings also suggest that deficiencies in, or impairment of, circulating ChAT may contribute to the pathogenesis of hypertension. Reduced ChAT expression in circulating lymphocytes and lymphoid organs has been previously linked to hypertension (Fujimoto et al. 2001).

In addition to the endothelium, plasma ACh has access to other cells which express ACh receptors. For example, erythrocytes express muscarinic receptors which, when activated by ACh, increase nitric oxide levels (Carvalho et al. 2004). Additionally, platelets, and both T and B lymphocytes respond to ACh raising the possibility of autocrine and paracrine regulation of immune function (Schedel et al. 2011; Fujii et al. 2017b). These and other biological activities distinct from those of the endothelium which are potentially modulated by circulating concentrations of ACh will require additional study to determine whether significant adverse effects occur following a change in plasma ChAT concentration.

In these acute experiments biological half-life following enzyme administration is relatively short lived, lasting several hours. The half life will need to be more quantitatively assessed following repeated dosing and the profile of blood pressure response followed to determine whether administration of unmodified ChAT could be a viable pharmaceutical intervention. More than likely, modifications to prolong half life or development of a delayed release formulation will be required. Notably, in these acute experiments pegylation of the enzyme, one of a variety of commonly used biochemical modifications to increase circulating half life of short lived compounds, results in retained enzymatic activity which provided moderately longer-lasting decrease in blood pressure than the unmodified enzyme. Finally, evalaution of ChAT administraton with currently utilized anti-hypertensives which target the underlying pathophysiology, particularly angiotensin converting enzyme inhibitors or angiotensin receptor blockers would be important to undertake.

In conclusion, our studies show that systemic administration of recombinant ChAT to mice with angiotensin II-induced hypertension significantly, dose-dependently and acutely decreases mean arterial pressure. ChAT-induced attenuation of blood pressure is reversed by inhibiting nitic oxide production, indicating that ChAT administration reduces blood pressure through nitic oxide-dependent vasodilation. Administration of PEG-ChAT to hypertensive mice decreased mean arterial pressure with a significantly longer response duration as compared to ChAT. The possible role of pegylation to improve the efficacy of ChAT as shown in the current study, should be thoroughly examined as a strategy to modify the half-life and efficacy of ChAT. These findings are of interest for the development of ChAT as a novel therapeutic for established hypertension.

## Supplementary Information

**Additional file 1: Figure S1.** ChAT administration decreases SBP and DBP. **A** Change in normalized SBP in the 3 h period after ChAT administration. SBP is normalized to the 30-min period before ChAT or vehicle administration. After normalization, area under the curve is calculated from a baseline of 100%. Negative values correspond to decrease in SBP. n = 7–11. *p < 0.05, one-way ANOVA with Dunnett’s correction. **B** Change in normalized DBP in the 3 h period after ChAT administration. DBP is normalized to the 30-min period before ChAT or vehicle administration. After normalization, area under the curve is calculated from a baseline of 100%. Negative values correspond to decrease in DBP. Data are represented as individual mouse data points, mean ± SEM. n = 7–11. *ns* not significant one-way ANOVA with Dunnett’s correction. **Figure S2.** ChAT administration increases heart rate. Change in heart rate after ChAT administration. Heart rate is averaged over the 30 min pre-administration. Change is calculated as the pre-administration baseline subtracted from the average heart rate in the 3 h post-administration period. Data are represented as individual mouse data points, mean ± SEM. n = 5–11, *p < 0.05, t-test. **Figure S3.** ChAT administration does not affect body temperature. Change in body temperature after ChAT administration. Body temperature is averaged over the 30 min pre-administration. Change is calculated as the pre-administration baseline subtracted from the average body temperature in the 3 h post-administration period. Data are represented as individual mouse data points, mean ± SEM. n = 5–11, *ns* not significant, t-test. **Figure S4.** ChAT administration increases activity levels. Change in activity after ChAT administration. Activity is averaged over the 30 min pre-administration. Change is calculated as the pre-administration baseline subtracted from the average activity in the 3 h post-administration period. Data are represented as individual mouse data points, mean ± SEM. Vehicle n = 5–11, *ns* not significant, t-test. **Figure S5.** PEGylation increases molecular weight without decreasing specific activity. **A** SDS-PAGE of purified recombinant human ChAT with and without PEGylation. With PEGylation, molecular weight increases by approximately 20 kDa. **B** comparison of specific activity of ChAT and PEG-ChAT, as determined by colorimetric activity assay. Data represented as one assay performed in triplicate. ****p < 0.0001, one-way ANOVA with Dunnett’s correction. **Figure S6.** PEG-ChAT administration decreases both SBP and DBP. **A** change in normalized SBP in the 3 h period after PEG-ChAT administration. SBP is normalized to the 30-min period before PEG-ChAT (0.1 or 1 mg/kg) or vehicle administration, and area under the curve is calculated from a baseline of 100%. Negative values correspond to decrease in SBP. Data are represented as individual mouse data points, mean ± SEM. n = 7–11 per group. ***p < 0.001, one-way ANOVA with Dunnett’s correction. **B** change in normalized DBP in the 3 h period after PEG-ChAT administration. SBP is normalized to the 30-min period before PEG-ChAT (0.1 or 1 mg/kg) or vehicle administration, and area under the curve is calculated from a baseline of 100%. Negative values correspond to decrease in DBP. Data are represented as individual mouse data points, mean ± SEM. n = 7–11 per group. **p < 0.01, one-way ANOVA with Dunnett’s correction. **Figure S7.** PEG-ChAT administration does not induce long-term changes in heart rate, body temperature, or activity levels. **A** HR, **B** temperature and **C** activity in the 24 h period post PEG-ChAT administration. Gray background indicates dark cycle. Data are averaged every hour and represented with lines as group mean ± SEM. *ns* not significant, two-way ANOVA with Sidak’s multiple comparisons test. **Figure S8.** PEG-ChAT administration during dark cycle induces decreases in SBP and DBP in the following light cycle. **A** mean SBP, **B** mean DBP in light cycle. PEG-ChAT was administered at the middle of the dark cycle (6 h), and MAP is averaged 6–18 h post-PEG-ChAT administration. Data are represented as individual mouse data points, mean ± SEM. Vehicle: n = 12, PEG-ChAT 0.1 mg/kg: n = 20, PEG-ChAT 1 mg/kg: n = 21, *p < 0.05, ***p < 0.001, ****p < 0.0001, one-way ANOVA with Dunnett’s correction.

## Data Availability

The datasets used and/or analysed during the current study are available from the corresponding author on reasonable request.

## Abbreviations

BP: Blood pressure
SBP: Systolic blood pressure
DBP: Diastolic blood pressure
MAP: Mean arterial blood pressure
ChAT: Choline acetyltransferase
NO: Nitric oxide
PEG: Polyethylene glycol
EDRF: Endothelium derived relaxation factor
T_ChAT_: T-choline acetyltransferase
AngII: Angiotensin II
HR: Heart rate
L-NAME: Nω-nitro-l-arginine methyl ester
