# Supplementary material for: Systemic administration of choline acetyltransferase decreases blood pressure in murine hypertension
Source: Mol Med. 2021 Oct 21;27:133. doi: 10.1186/s10020-021-00380-6 (PMC8529785; doi:10.1186/s10020-021-00380-6)
Supplement: Supplementary file 1 — Additional file 1: Figure S1. ChAT administration decreases SBP and DBP. A Change in normalized SBP in the 3 h period after ChAT administration. SBP is normalized to the 30-min period before ChAT or vehicle administration. After normalization, area under the curve is calculated from a baseline of 100%. Negative values correspond to decrease in SBP. n = 7–11. *p < 0.05, one-way ANOVA with Dunnett’s correction. B Change in normalized DBP in the 3 h period after ChAT administration. DBP is normalized to the 30-min period before ChAT or vehicle administration. After normalization, area under the curve is calculated from a baseline of 100%. Negative values correspond to decrease in DBP. Data are represented as individual mouse data points, mean ± SEM. n = 7–11. ns not significant one-way ANOVA with Dunnett’s correction. Figure S2. ChAT administration increases heart rate. Change in heart rate after ChAT administration. Heart rate is averaged over the 30 min pre-administration. Change is calculated as the pre-administration baseline subtracted from the average heart rate in the 3 h post-administration period. Data are represented as individual mouse data points, mean ± SEM. n = 5–11, *p < 0.05, t-test. Figure S3. ChAT administration does not affect body temperature. Change in body temperature after ChAT administration. Body temperature is averaged over the 30 min pre-administration. Change is calculated as the pre-administration baseline subtracted from the average body temperature in the 3 h post-administration period. Data are represented as individual mouse data points, mean ± SEM. n = 5–11, ns not significant, t-test. Figure S4. ChAT administration increases activity levels. Change in activity after ChAT administration. Activity is averaged over the 30 min pre-administration. Change is calculated as the pre-administration baseline subtracted from the average activity in the 3 h post-administration period. Data are represented as individual mouse data points, mean ± SE [file 10020_2021_380_MOESM1_ESM.pdf]

Additional Figure 1

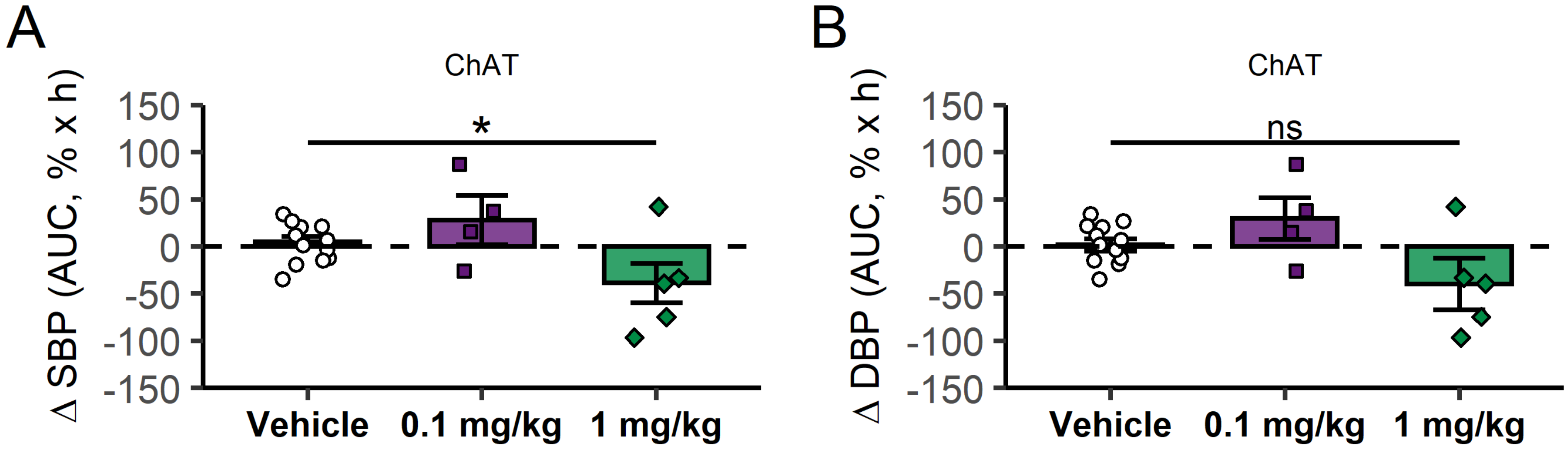

Additional Figure 2

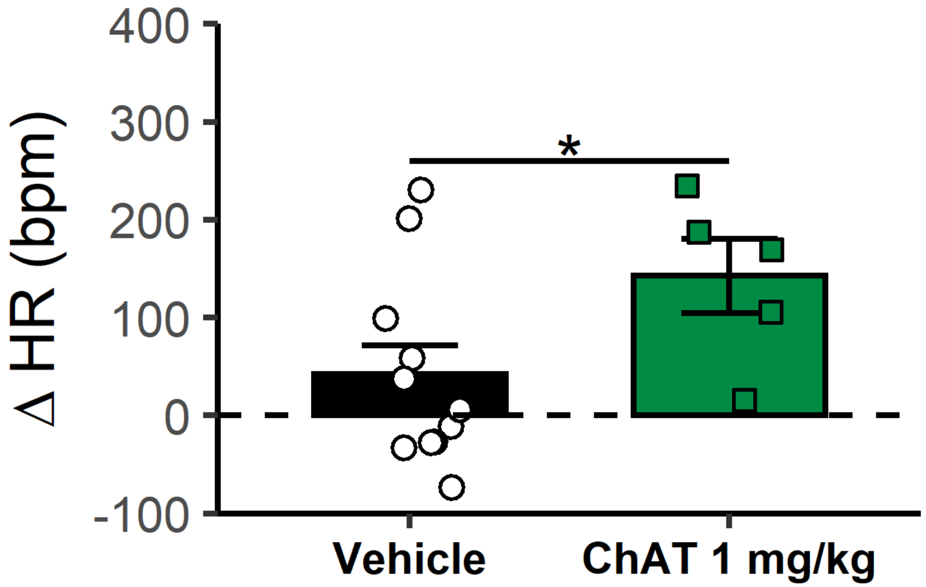

Additional Figure 3

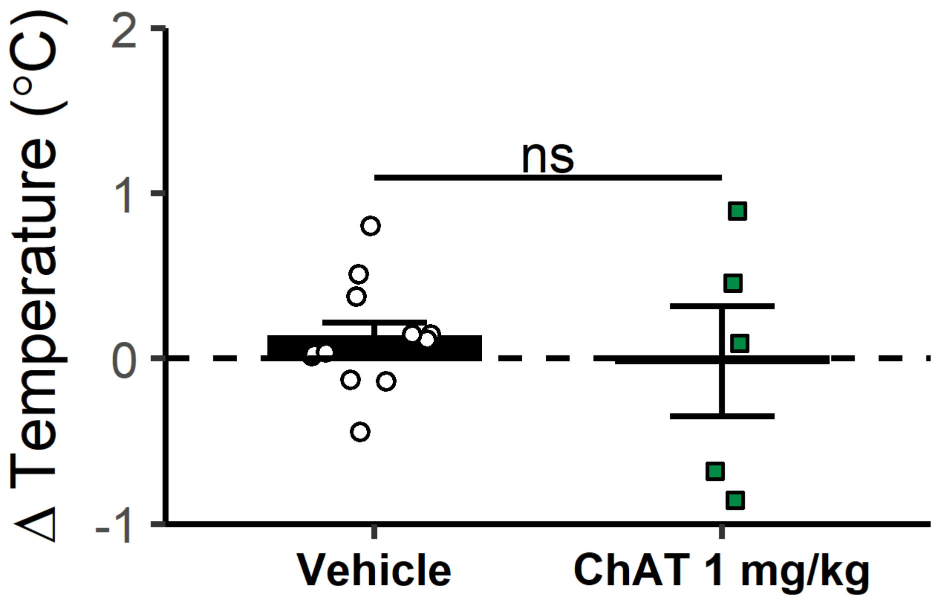

Additional Figure 4

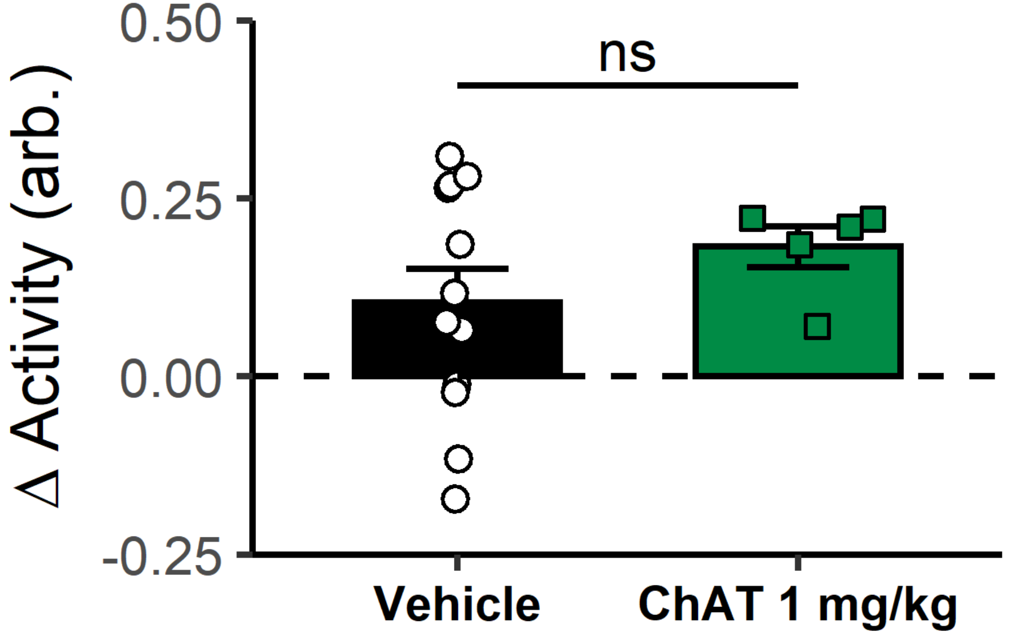

Additional Figure 5

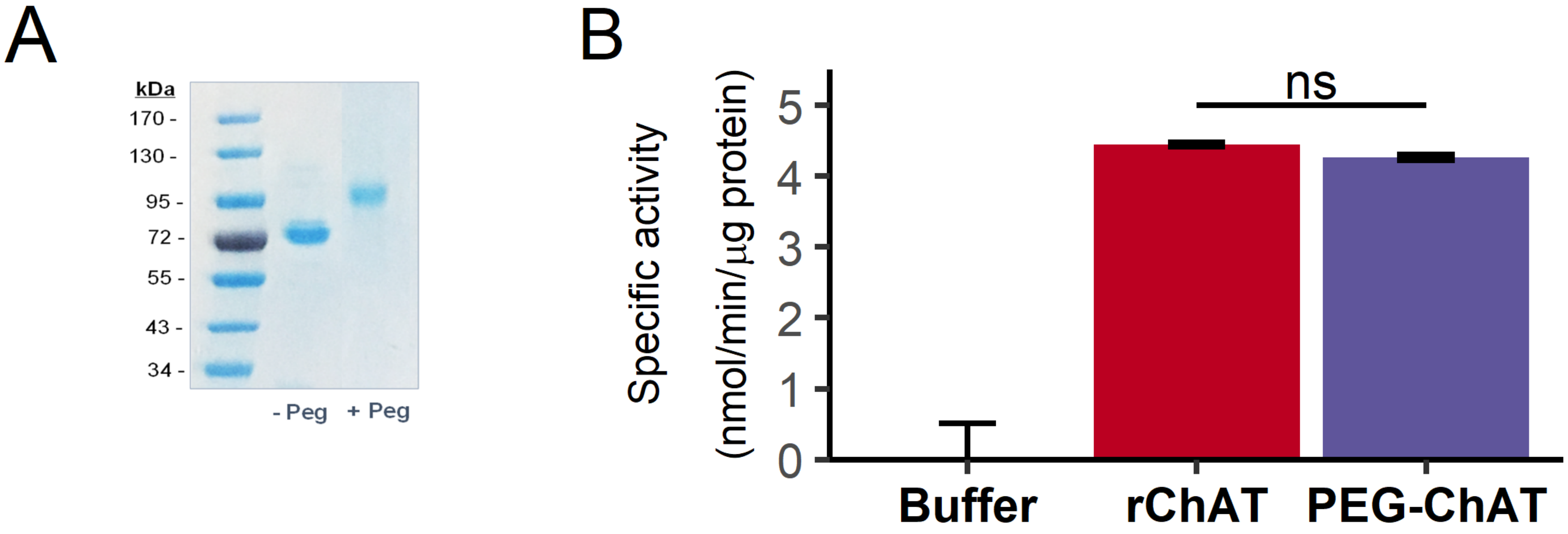

Additional Figure 6

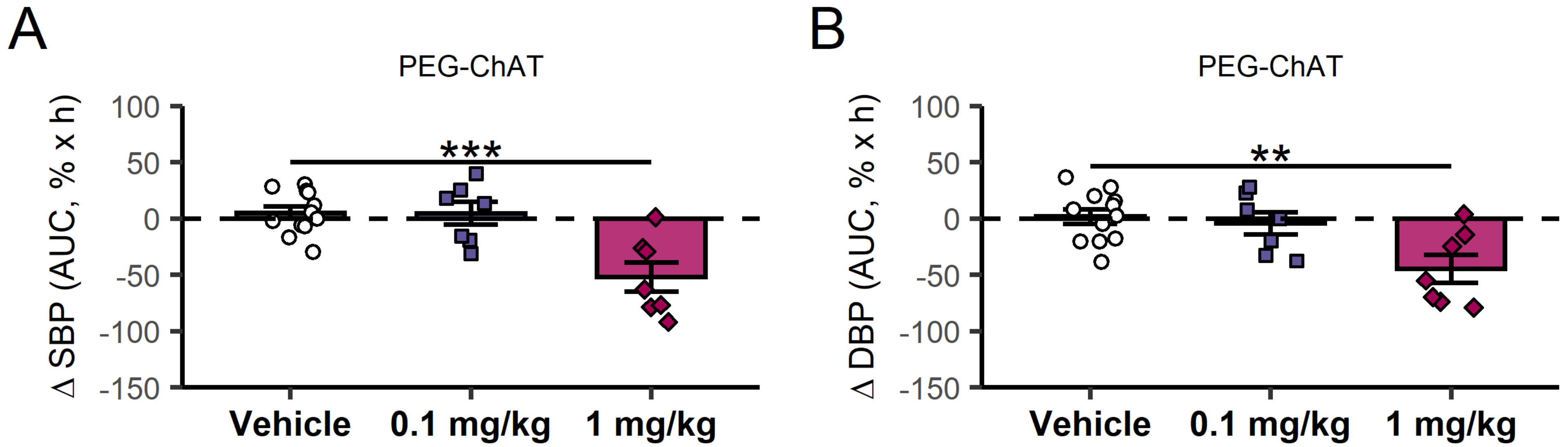

Additional Figure 7

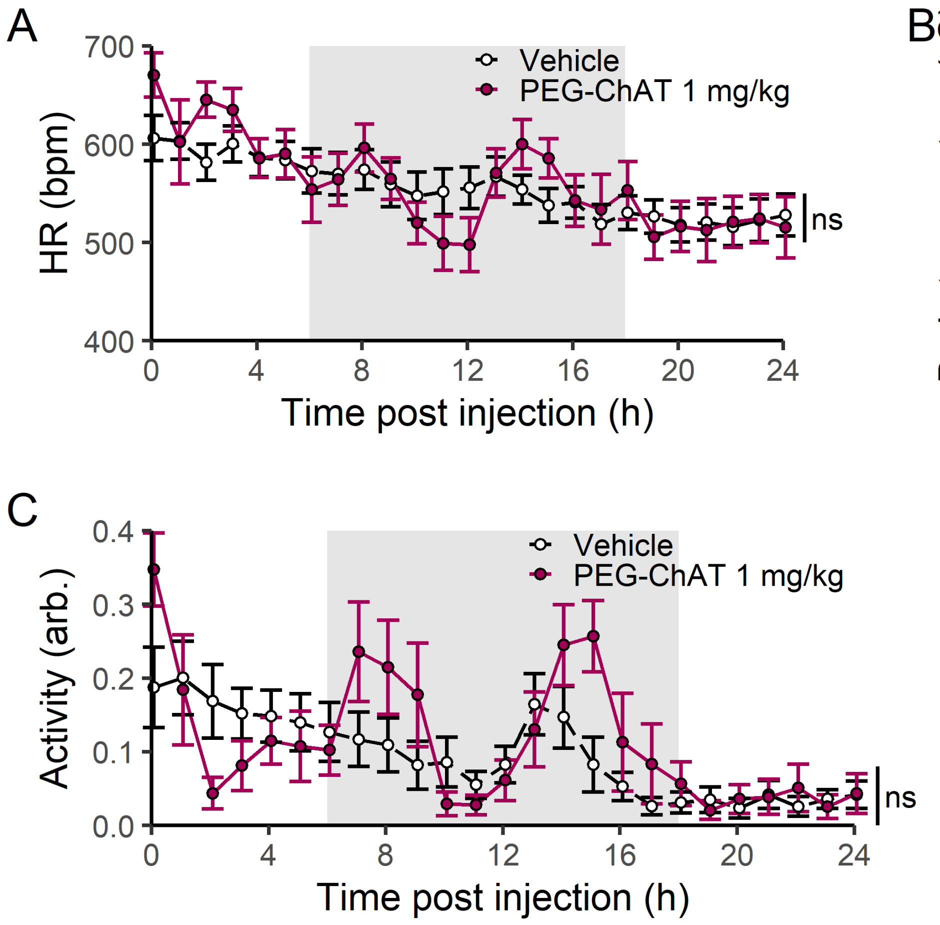

Additional Figure 8

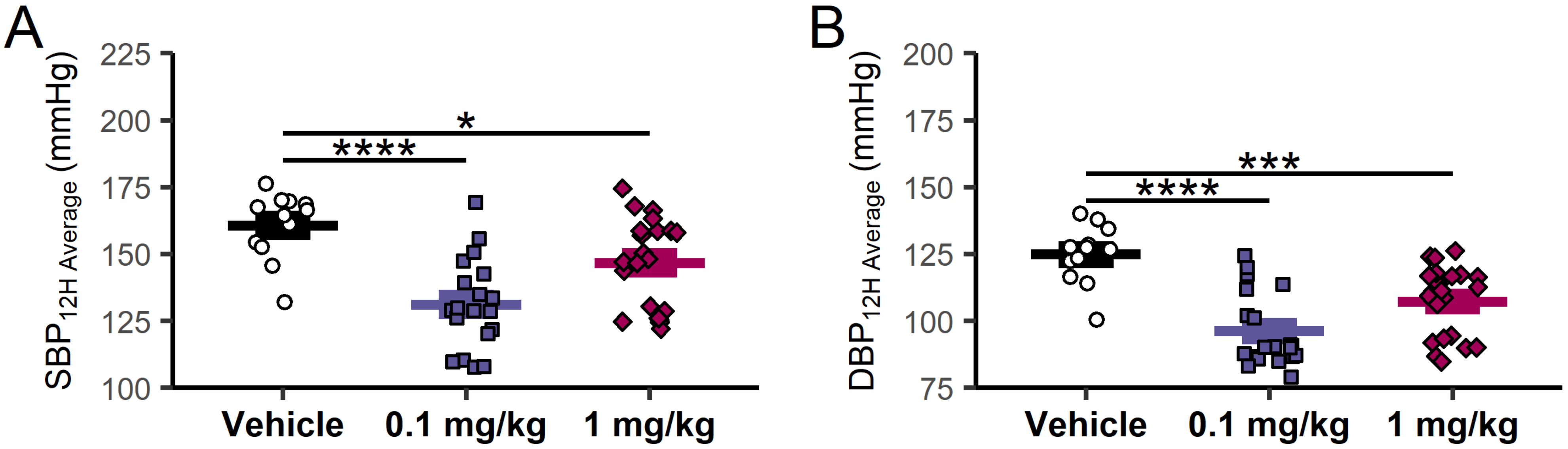

Table 1

MAP, mmHg		Mean	95% CI	Range
Normotensive	Light cycle	101	(99.6 - 102.5)	92 - 120
	Dark cycle	109	(108.3 - 110.4)	102 - 123
Hypertensive	Light cycle	119	(115.8 - 122.5)	98 - 150
	Dark cycle	136	(133.6 - 139.0)	111 – 153

SBP, mmHg		Mean	95% CI	Range
Normotensive	Light cycle	120	(118.3 - 120.9)	112 - 134
	Dark cycle	128	(126.7 - 129.3)	120 - 140
Hypertensive	Light cycle	144	(139.7 - 147.8)	119 - 172
	Dark cycle	163	(159.2 - 166.4)	131 - 185

DBP, mmHg		Mean	95% CI	Range
Normotensive	Light cycle	92	(90.0 - 93.5)	82 - 112
	Dark cycle	100	(98.8 - 101.2)	92 - 114
Hypertensive	Light cycle	107	(103.5 - 110.1)	87 - 139
	Dark cycle	123	(120.4 - 125.7)	102 - 141
